# Digestive Inflammation: Role of Proteolytic Dysregulation

**DOI:** 10.3390/ijms22062817

**Published:** 2021-03-10

**Authors:** Vincent Mariaule, Aicha Kriaa, Souha Soussou, Soufien Rhimi, Houda Boudaya, Juan Hernandez, Emmanuelle Maguin, Adam Lesner, Moez Rhimi

**Affiliations:** 1 Microbiota Interaction with Human and Animal Team (MIHA), Micalis Institute, AgroParisTech, Université Paris-Saclay, INRAE, F-78350 Jouy-en-Josas, France; vincent.mariaule@gmail.com (V.M.); aicha.kriaa@gmail.com (A.K.); souha.soussou.1994@gmil.com (S.S.); soufienrhimi@yahoo.fr (S.R.); boudayahouda12@gmail.com (H.B.); emmanuelle.maguin@inrae.fr (E.M.); 2 Department of Clinical Sciences, Nantes-Atlantic College of Veterinary Medicine and Food Sciences (Oniris), University of Nantes, 101 Route de Gachet, 44300 Nantes, France; juan.hernandez@oniris-nantes.fr; 3 Faculty of Chemistry, University of Gdansk, Wita Stwosza 63, PL80-308 Gdansk, Poland; adam.lesner@ug.edu.pl

**Keywords:** gut microbiota, protease, serpin, digestive inflammation, holobiont

## Abstract

Dysregulation of the proteolytic balance is often associated with diseases. Serine proteases and matrix metalloproteases are involved in a multitude of biological processes and notably in the inflammatory response. Within the framework of digestive inflammation, several studies have stressed the role of serine proteases and matrix metalloproteases (MMPs) as key actors in its pathogenesis and pointed to the unbalance between these proteases and their respective inhibitors. Substantial efforts have been made in developing new inhibitors, some of which have reached clinical trial phases, notwithstanding that unwanted side effects remain a major issue. However, studies on the proteolytic imbalance and inhibitors conception are directed toward host serine/MMPs proteases revealing a hitherto overlooked factor, the potential contribution of their bacterial counterpart. In this review, we highlight the role of proteolytic imbalance in human digestive inflammation focusing on serine proteases and MMPs and their respective inhibitors considering both host and bacterial origin.

## 1. Introduction

The incidence of inflammatory bowel diseases (IBD) is rising in the Western world, affecting millions of patients in the US and Europe. Over the past decades, developing countries in Asia, South America and the Middle East reported the emergence of IBD, thus highlighting its evolution as a global disease [1]. Modern treatments are significantly improving the quality of life of patients; however, despite advances in the therapeutic field, treatment failure is common [2]. The etiology of IBD remains incompletely comprehended and seems to result from the interaction of genetic and environmental factors [3]. In the area of host–microbe interactions in the gastrointestinal (GI) tract, environmental factors can modify the diversity and composition of the intestinal microbiota, which, for genetically predisposed individuals, attends a deterioration of the epithelial barrier integrity and triggers abnormal immune response [4].

The intestinal mucosa not only acts as a physical barrier preventing the entry of microorganisms or harmful components from the lumen into the blood circulation but also allows dietary nutrients’ absorption. Moreover, the GI tract hosts hundreds of trillions of microbes and is exposed to high levels of proteases. An important part of the literature has raised the key role of proteases in maintaining GI homeostasis, and their upregulation causes tissue damage and inflammation [5,6]. Recent studies have established the association between increased serine protease activity and IBD pathogenesis [7,8]. Due to their implication in tissue remodeling through their ability to degrade extracellular matrix (ECM) components and their immunomodulating effects [9], matrix metalloproteinases (MMPs) are considered key actors of IBD pathogenesis and its related complications such as fistula and fibrosis [10]. Under healthy conditions, the proteolytic activity of serine proteases and MMPs is tightly controlled by their respective protease inhibitors due to their implication in many biological processes. Dysregulation of this balance contributes to IBD pathophysiology [5,11]. In this review, we present a summary of the current literature on the proteolytic imbalance in digestive inflammation focusing on serine proteases and MMPs originating from host and gut microbiota. We also highlight synthetic inhibitors that have reached the clinical trial phase as a candidate for IBD treatment.

## 2. Association between Proteolytic Enzymes and Digestive Inflammation

Serine proteases and metalloproteinases constitute two protease subfamilies, showing distinct structural features. In the case of metalloproteinases during peptide cleavage, the nucleophile attack is mediated by a water molecule in the presence of a Zn^2+^ divalent ion. However, the serine proteases form a covalent complex between the peptide and the catalytic serine residue upon the nucleophilic attack. Several reports have stressed the key role of these proteases in digestive inflammation.

### 2.1. Metalloproteases

MMPs are endoproteases containing a conserved zinc-binding motif in their catalytic site. This enzyme family shares a common domain organization consisting of a propeptide, a catalytic domain, a hinge region (linker) and a hemopexin domain. They can degrade components of the ECM, mediating its homeostasis. The cellular source of MMPs encompasses a wide range of cell types, including epithelial cells, macrophages, leukocytes, neutrophils and myofibroblasts. Investigation studies on MMP substrates’ specificity have unveiled the diversity of molecules cleaved by MMPs, including chemokines, cytokines, growth factors and receptors, thus shedding light on the involvement of MMPs in other biological processes such as angiogenesis, immunity and inflammatory response. Consistent with their role as key regulators, MMP activity is tightly regulated at several levels, from gene expression, activation to inhibition by specific inhibitors. MMPs inhibition will be discussed more specifically in a dedicated section of the present review.

Dysregulation in MMP expression and activity has been associated with several pathologic processes such as cancers, cardiovascular diseases, musculoskeletal disorders and chronic inflammation. In the context of IBD, many MMPs are found to be upregulated; for instance, MMP-1, -2, -3, -7, -8, -9, -10, -12 and -13. In patients with ulcerative colitis (UC), a correlation between MMP-1 expression in colonic mucosa and severity of clinical symptoms has been established [12]. In line with previous studies that have identified MMP-2 overexpression in the colonic mucosa of UC patients, Jakubowska et al. [13] demonstrated weak MMP-2 expression in infiltrative inflammatory cells and strong expression in the glandular epithelium of UC and Crohn’s disease (CD) patients. Matsuno et al. [14] established that the level of matrilysin (MMP-7) expression in epithelial cells at the edge of the ulcer of UC patients ties in with the disease activity. Conversely, stromelysin-1 (MMP-3) expression levels in epithelial and stromal cells were not different between patients with mild and those with severe inflammation. Immunostaining of mucosal samples from IBD patients revealed the enhanced expression of MMP-13, another member of the collagenase group of MMPs, which has been positively linked to histological inflammation scores [15]. Indeed, MMP-13 modulates intestinal permeability through the shedding of the transmembrane-bound tumor necrosis factor (TNF), thus releasing active soluble TNF [16]. This release has two effects: (i) it induces caveolin-dependent endocytosis, which results in tight junction (TJ) destabilization, and (ii) it stimulates the expression and secretion of mucin by goblet cells, which eventually cause endoplasmic reticulum (ER) stress, resulting in mucus depletion and leading to increased interactions between bacteria and intestinal epithelial and Paneth cells. Additionally, moderate protection to dextran sulfate sodium (DSS)-induced colitis was observed in MMP13^−^/^−^ mice compared to MMP13^+^/^+^ mice [16]. In past years, many studies have focused on gelatinase B (MMP-9) as a novel therapeutic target for IBD treatment as a result of the association between its expression and disease development [17,18]. Al-Sadi et al. [19] demonstrated that MMP-9 causes an increase in intestinal tight-junction permeability via the p38 kinase signaling pathway, upregulating myosin light-chain kinase (MLCK) gene expression. Recent work [20] suggests that MMP-9 upregulation is a consequence of intestinal inflammation, which is in contradiction with a previous investigation that suggests its causative implication in an experimental model of colitis [21]. Collagen degradation by MMPs is an important factor in neutrophilic inflammation in IBD [22]. Neutrophil recruitment to sites of infection is usually mediated by the CXCL8 chemokine. Yet, proline-glycine-proline (PGP) peptide resulting from collagen degradation is also a neutrophil chemoattractant. Three enzymes are involved in its production: MMP-8, MMP-9 and prolyl endopeptidase (PE). Intestinal tissues from IBD patients have increased levels of MMP-8, MMP-9, PGP and its acetylated version (N-Ac-PGP), and PE levels show no difference compared to control. In mice with dextran sulfate sodium (DSS)-induced colitis, PGP neutralization results in a significant reduction of the disease activity index (DAI) score and infiltrating neutrophils, emphasizing its pathophysiological role in neutrophilic inflammation. Considering that (i) in vitro PGP induces the release of MMP-9 and CXCL8 from neutrophils and (ii) neutrophils from IBD patients secrete more MMP-8 and MMP-9 under unstimulated conditions and have increased migration capabilities toward CXCL8 than neutrophils from healthy patients, they form elements of a vicious circle of sustained neutrophilic inflammation in IBD. Recently, the contribution of MMP-10 and, to some extent, MMP-9 and -7 to CD, has been highlighted through the identification of their action on programmed death-ligand 1 (PD-L1), an immune regulatory molecule present in myofibroblasts (MFs) [23]. In healthy conditions, MFs can suppress T-helper 1 (Th1)- and T-helper 17 (Th17)-type responses through the presence of PD-L1 molecules at their surface. In CD conditions, increased MMP-10 expression is linked to reduced levels of membrane-bound PD-L1 (mPD-L1) and a rise of the soluble form of PD-L1, leading to an impairment of the suppressing ability of MF on Th1 and Th17 activities. Levels of mPD-L1 of CD-MF are reinstated upon MMP-10 inhibition as well as their subsequent suppressing abilities. Although a strong increase in mPD-L1 levels is observed when inhibiting MMP-7 and MMP-9, the MF-mediated T-helper suppression function is only partially restored. The protective effect of MMP-19 over colitis seems to originate from its capacity to control neutrophils and macrophage migration to wounded mucosa, possibly through the processing of the chemokine domain of fractalkine (CX3CL1) [24]. In a DDS-induced model of colitis, MMP-19^−^/^−^ mice show increased susceptibility and exacerbation of colitis reflected by a reduced survival rate, severe tissue destruction, increased levels of colonic and a plasmatic level of proinflammatory modulators and failure to resolve inflammation. In addition, a delay in the infiltration of neutrophils into the colon and reduced migration of macrophages is observed.

Of note, the MMP field of action may be extended to the primary nonresponsiveness of IBD patients to anti-TNF treatments [25]. Indeed, Barberio et al. [26] established that MMP-3 serum levels of IBD patients treated with infliximab are higher in nonresponders compared to responders. This suggests that MMP-3 serum levels may represent an early predictive marker of response to infliximab. In line with these results, three anti-TNF agents (infliximab, adalimumab and etanercept) are degraded by MMP-3 and MMP-12 in vitro, notably by the removal of their Fc region. While infliximab and adalimumab maintained their ability to neutralize TNF after MMP treatment, etanercept lost its neutralization capability. Notwithstanding, loss of the Fc region may still have clinically relevant consequences in vivo as some immunologic properties such as antibody-dependent, cell-mediated cytotoxicity and complement activation require an Fc region. Further in vivo investigations are required to decipher the real impact of MMPs on the nonresponsiveness to anti-TNF treatment mechanisms in IBD patients.

**Figure 1:**
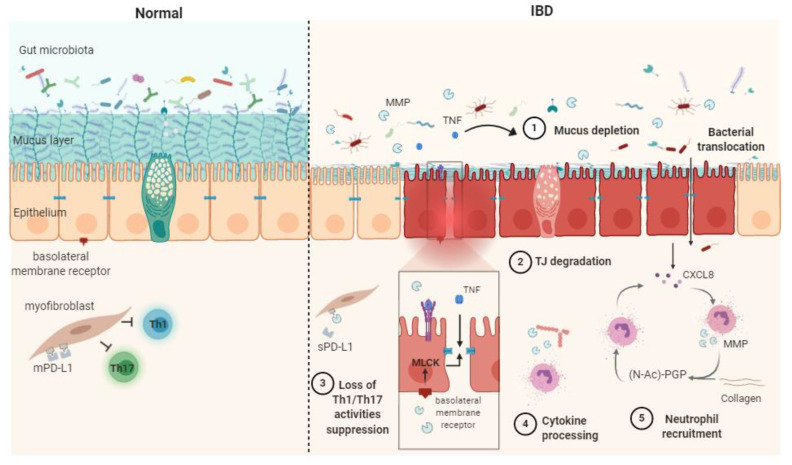
Schematic representation of matrix metalloproteases (MMPs) mechanisms of action in healthy contet (**Normal**) and in inflammatory bowel disease (**IBD**) pathophysiogenesis. The release of soluble TNF-α resulting from the shedding of membrane-bound TNF by MMPs causes mucus depletion (**1**) and tight-junction destabilization (**2**), leading to increased epithelial permeability and bacterial translocation. The abrogation of myofibroblast ability in suppressing Th1/Th17 occurs after the MMP processing of membrane-bound PD-L1 to produce soluble PD-L1 (**3**). Cytokine processing contributes to inflammation processes (**4**). PGP generation through collagen degradation by MMPs induces neutrophil transmigration and stimulates neutrophil MMP and CXCL8 secretion, therefore sustaining the inflammatory context (**5**). CXCL8: (C-X-C motif) ligand 8, MLCK: myosin light-chain kinase, PD-L1: programmed death-ligand 1, mPD-L1: membrane-bound PD-L1, sPD-L1: soluble PD-L1, (N-Ac)-PGP: (N-acetyl)-proline-glycine-proline, TJ: tight junction, TNF: tumor necrosis factor α.

In summary, the dysregulation of MMPs can contribute to IBD via five main processes: cytokine processing, mucus depletion, tight-junction destabilization, neutrophil recruitment and stimulation and Th1/Th17 response (Figure 1).

### 2.2. Serine Proteases

The GI tract is regularly exposed to high levels of proteolytic enzymes from both host and enteric bacteria [27]. From the host side, these proteases can be released either by resident or infiltrating cells. Among infiltrating immune cells, neutrophils constitute a prime source of serine proteases. Their granules harbor significant amounts of elastase (HNE), proteinase 3 (PR3) and cathepsin G (catG), which are secreted upon inflammation [28]. Serine proteases can also originate from mast cells, which release tryptase, chymase, catG and granzyme B [29]. Indeed, increased serine protease activity has been detected in both tissue biopsies and fecal samples from IBD patients [7,8,11,30]. As major components of the neutrophil proteolytic repertoire, these proteases contribute to the inflammatory response by cleaving junctional proteins, activating protease-activated receptors (PARs) and processing cytokines and chemokines that are in charge of the recruitment and activation of immune cells to the site of inflammation [31]. Neutrophil elastase and catG, for instance, cleave the vascular endothelial cadherin occurring at cellular junctions and thus contribute to leukocyte transmigration to inflammatory sites [32,33]. Neutrophil proteases also participate in MMP regulation notably through the activation of pro-MMP2 by catG, PR3 and HNE [34] and the inactivation of TIMP-1 by the HNE [35]. Increased levels of HNE have been previously detected in plasma and the colonic mucosa from IBD patients and are therefore explored as a potential biomarker for IBD [36,37]. Such uncontrolled activity was shown to elicit detrimental effects and drive inflammation in a murine model [38]. CatG also activates PAR4 and elicits the disruption of the epithelial barrier integrity [39]. It is assumed that such effects were associated with MLCK activation, myosin light-chain (MLC) phosphorylation and subsequent TJ destabilization. CatG and PR3 also cleave chemokines such as CXCL5 and CXCL8, thus contributing to higher chemotactic activity toward neutrophils [40]. Other examples include thrombin, which showed a 100-fold increase in activity in colonic biopsies from IBD patients compared with healthy controls [7]. Such activity has been suggested to be derived from the intestinal epithelium and/or the recruitment and activation of prothrombin at the damaged sites following vascular lesions. Active thrombin has been shown to mediate claudin-5 disassembling and increase vascular permeability in vivo [41]. The same response was also induced by PAR1 agonists, suggesting a key role for PAR1 activation in the proinflammatory effects of thrombin. PAR1 is the prototype receptor of thrombin [42]. PAR4 can also be cleaved by thrombin, and quite recently, thrombin signaling through PAR2 activation has been uncovered [43]. Note that the inhibition of colonic thrombin by the intracolonic injection of dabigatran, a thrombin inhibitor, in 2,4,6-trinitrobenzenesulfonic acid (TNBS)-induced colitis in rats, resulted in a significant reduction of the inflammatory parameters. Mucosal mast cell chymase was also shown to evoke proinflammatory effects as it alters the distribution of tight-junction-associated proteins such as ZO-l and occludins and increases epithelial permeability [44]. The role of this enzyme is not limited to enhanced gut permeability and includes MMPs activation [45]. Indeed, chymase can convert pro-MMP-9 to its active form MMP-9 in vitro and therefore plays a role in extracellular matrix remodeling [45]. A recent study shows the implication of tryptase in promoting IBD-induced intestinal fibrosis by activating the PAR-2/Akt/mTOR pathway in fibroblasts [46]

**Figure 2:**
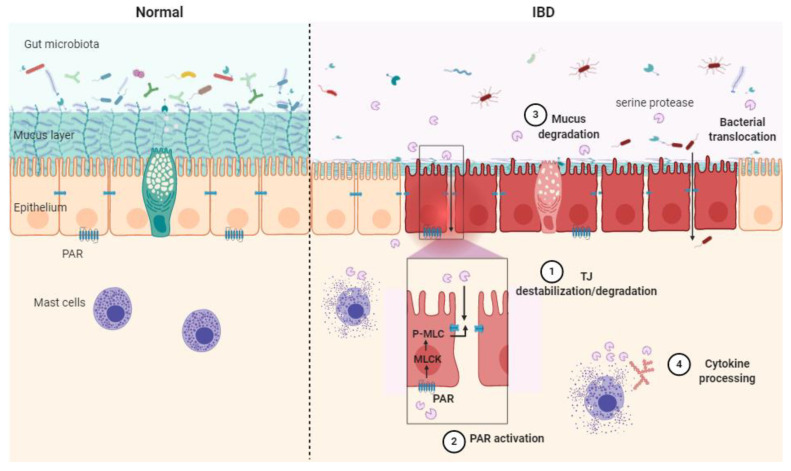
Schematic illustration of the serine protease mode of action in healthy context (**Normal**) and in inflammatory bowel disease (**IBD**) pathology. Epithelial barrier impairment is associated with serine protease action on the tight junction through direct cleavage. (**1**) Indirect destabilization deriving from protease-activated receptor (PAR) activation (**2**), mucus degradation (**3**) and cytokine processing (**4**). MLC: myosin light-chain, P-MLC: phosphorylated MLC, MLCK: myosin light-chain kinase, PAR: protease-activated receptor, TJ: tight junction.

Serine protease contribution to IBD can be described through four main mechanisms: TJ destabilization/degradation, mucus degradation, PAR activation and cytokine processing (Figure 2).

### 2.3. Role of Bacterial Proteases in Digestive Inflammation

The role of microbial proteases in the gut has been largely dismissed, partly due to limited tools setting apart host proteases from their microbial counterparts. Earlier studies have defined a significant contribution of bacterial proteases to proteolysis in the human large intestine [47]. Most identified proteases belong to *Bacteroides*, *Streptococcus* and *Clostridium* species [48]. Seeing that proteases are often studied as virulence factors, pathogen-derived proteases have mostly been explored for their effects in the GI tract. Such proteases have been described as key factors in (i) helping the bacterium to successfully compete with resident microbiota during infection and (ii) promoting bacterial fitness and survival under hostile conditions. Years ago, high-temperature serine protease A (HtrA) was defined as a key virulence factor of *Listeria monocytogenes*. *L. monocytogenes* is a facultative pathogen that has been shown to actively invade macrophages and epithelial cells as well as other neighboring host cells [49]. The lack of HtrA expression results in the impaired growth of such a bacterium under stressful conditions, including acidic pH or oxidative stress [50,51]. Additionally, an *L. monocytogenes* HtrA mutant revealed a reduced ability to form biofilms and was dimmed for virulence in mice [52]. Recently, a new presumed role of HtrA has been highlighted in listerial replication during infection, thus outlining the relevance of these chaperone serine proteases in bacterial infection [53]. The contribution of HtrA proteases to bacterial virulence has been explored in many other pathogens, including *Campylobacter jejuni*, *Helicobacter pylori* and *Borrelia burgdorferi* [54,55,56]. The main role of HtrA is related to protein quality control and the degradation of misfolded proteins to enhance bacterial fitness under hostile conditions. HtrA is also involved in the processing of tight junctional proteins, thereby leading to the disruption of epithelial barrier integrity [54,55,56]. Other bacteria, including intestinal adherent and invasive *Escherichia coli* (AIEC), most likely secrete serine proteases to invade the mucous layer. A recently described protease produced by AIEC, known as VAT-AIEC, has been shown to contribute to gut colonization in a murine model by enhancing the expansion of bacteria through the mucous layer and adhesion to intestinal epithelial cells [57]. Besides enteric pathogens, nonvirulent bacteria also produce an extremely diverse repertoire of proteolytic enzymes that might contribute to gut inflammation. Subtilisin, a serine protease produced by the nonpathogenic *Bacillus subtilis*, has been shown to elicit plasma clotting and venous thromboembolism by proteolytically converting prothrombin (ProT) into active prethrombin-2 (σPre2) [58]. Venous thromboembolism is a known complication in patients with inflammatory disorders such as IBD that has been associated with significant morbidity and mortality [59,60].

The production of MMP by intestinal bacteria has been described as well. Strains of *Bacteroides fragilis*, for instance, produce a MMP that is able to cleave the ECM component E-cadherin [61], and *Bacteroides thetaiotaomicron* encodes putative proteases with similar homology [62]. E-cadherin plays critical roles in maintaining the integrity of the epithelium barrier, and the loss or reduction of this protein expression has been linked to gastrointestinal disorders [63,64]. *Clostridium perfringens* MMP can target components of the ECM such as gelatin, type IV collagen and mucin and effectively degrade the mucus barrier [65]. More recently, the commensal bacterium *Enterococcus faecalis* was shown to produce gelatinase that cleaves E-cadherin, promoting colonic barrier impairment, thus increasing colitis severity in mice [66]. As proteases exhibit broad and pleiotropic effects, one could hypothesize that their microbial counterparts may have similar effects and could influence inflammation, wound healing, mucus cleavage, matrix remodeling, etc. As such, microbial proteolytic balance could be considered a promising contributor to gut homeostasis.

## 3. Protease Inhibition

### 3.1. Synthetic Protease Inhibitors

Increased expression of serine proteases (HNE, PR3, tryptase, catG, trypsin, chymotrypsin, chymase and thrombin) and MMP (MMP-2, -3, -9, -10, -12, -13, etc.) has been documented during digestive diseases, making the inhibition of these proteases a potential therapeutic avenue [5,67,68]. The last few years have brought several studies on the design of potent and highly selective synthetic inhibitors of serine proteases and MMPs to treat human diseases (Table 1). Although these engineered synthetic inhibitors are potential treatments of digestive diseases, more research in models of colitis is required before they can be practically applied.

**Table 1:** Recent synthetic inhibitors of serine proteases and matrix metalloproteases (MMPs) developed as potential therapeutic agents.

	Protease Inhibitor	Targets	Ki\IC50	References
**Matrix Metalloprotease Inhibitors**	Compound 10a	MMP-2	IC50 = 0.19 nM	[69]
	M219^hy^		Ki = 1.9 nM	[70]
	Compound 9a		IC50 = 9 nM	[71]
	Compound 8k	MMP-3	IC50 = 0.4 µM	[72]
	N-TIMP2_9_1_	MMP-9	Ki = 0.78 nM	[73]
	Compound 8		IC50 = 4.49 nM	[74]
	JNJ0966		IC50 = 440 nM	[75]
	Compound 16	MMP-10	IC50 = 24 nM	[76]
	Compound 4	MMP-12	Ki = 0.19 nM	[77]
	RXP470.1		IC50 = 0.24 nM	[78]
	Compound 4a		IC50 = 33 nM	[79]
	Compound 3		IC50 = 40 nM	[80]
	Compound 21k	MMP-13	IC50 = 0.0039 nM	[81]
	Compound 26c		IC50 = 0.0069 nM	[82]
	Compound 31f		IC50 = 0.036 nM	[83]
	Compound 35		IC50 = 0.071 nM	[84]
	Compound 34		IC50 = 0.36 nM	[85]
	Compound 15		IC50 = 1 nM	[86]
**Serine Protease Inhibitors**	Diazaborines	Elastase	-	[87]
	ER143	HNE	IC50 = 0.67 nM	[88]
	Compound 24		IC50 = 0.24 μM	[89]
	Bt-Val-Tyr-Asp-nValp(O-C_6_H_4_-4-Cl)_2_	PR3	Ki = 5.4 nM	[90]
	azapro-3		Ki = 1.5 µM	[91]
	keto-D-DYFRET		Ki = 1.7 µM	[92]
	Compound 1a	Tryptase	IC50 = 1.82 nM	[93]
	BMS 262084		IC50 = 4 nM	[94]
	Compound 12s + 4s		IC50 = 5.29 nM	[95]
	GTCnXSDPPICFPN	Cathepsin G	Ki = 1.6 nM	[96]
	carboxymethyl-BA		IC50 = 3.4 µM	[97]
	Compound 1	Trypsin	Ki = 0.02 nM	[98]
	Compound 7		Ki = 0.03 nM	[99]
	Compound 8		Ki = 0.16 nM	[99]
	Tat-loop		Ki = 0.607 µM	[100]
	F^8^-PPF-BBI	Chymotrypsin	Ki = 0.85 µM	[100]
	BF9-N17Y		-	[101]
	Compound 20	Chymase	Ki = 1.8 nM	[102]
	Compound 19		Ki = 2.2 nM	[102]
	Compound 5f		IC50 = 8.9 nM	[103]
	Compound 14s	Thrombin	IC50 = 3.23 nM	[104]
	Compound 12a		IC50 = 3.52 nM	[105]
	Compound 14m		IC50 = 3.71 nM	[104]
	Compound 12c		IC50 = 4.26 nM	[105]
	Compound 12c		IC50 = 10.94 nM	[106]

Hy: hydroxamate group.

#### 3.1.1. Synthetic MMP Inhibitors in Clinical Trials

Several studies have examined the potential of synthetic MMP inhibitors (MMPIs) in colitis models. Batimastat, a hydroxamic acid-based zinc MMPI, is also known as BB94. It is a potent broad-spectrum MMPI that has shown beneficial effects in reducing inflammation in rat experimental colitis [107]. However, this compound has poor solubility and generates mild toxicity and side effects, including abdominal pain [108]. Ilomastat (or GM6001), another hydroxamic acid derivative, is a broad-spectrum and potent inhibitor that has shown a protective effect on TNBS-induced colitis in rats by reducing MMP-1 level [109]. CGS-27023-A (MMI-270), a sulfonamide derivative that has a broad spectrum of inhibition for MMPs, has been shown to attenuate colonic mucosal injury in TNBS-induced colitis in rats by reducing MMP-2 and MMP-9 expression [110]. However, this compound was largely unsuccessful in a clinical trial due to its side effects such as muscle and joint pain [111]. Despite their beneficial effects in treating digestive inflammation, the above-mentioned broad-spectrum and nonselective MMPIs were largely unsuccessful in clinical trials because of their poor solubility and negative side and off-target effects. To resolve this issue, other inhibitors have been developed. Heimesaat et al. [112] reported that the selective gelatinase (MMP-2, MMP-9) inhibitor RO28-2653 ameliorated acute DSS-induced colitis in mice. Minocycline, a semisynthetic tetracycline, was tested in experimentally induced acute colitis in mice and was found to attenuate inflammation by blocking iNOS, MMP-2, -3, -9 and -13 expression in intestinal tissues [113,114].

Andecaliximab, a recombinant chimeric IgG4 monoclonal antibody directed against pro and active forms of MMP-9, is so far the only antibody that has reached the clinical trial stage. Despite promising results from preliminary and phase 1 clinical studies on the treatment of UC [18,115], clinical phase 2/3 performed on UC patients [116] and a clinical phase 2 on CD patients [117] concluded the lack of efficacy of andecaliximab. As suggested by de Bruyn et al. [118], several differences between phase 1 and phase 2/3 studies such as study endpoints, patient characteristics and the small number of patients actually treated subcutaneously with andecaliximab in the phase 1 study may partially explain these contradictory results.

#### 3.1.2. Synthetic Serine Protease Inhibitors in Clinical Trials

Contrary to other diseases such as pulmonary inflammation, only a few synthetic inhibitors have been tested in models of colitis to treat digestive diseases. In 2000, Onomura and coworkers [119] noted that the thrombin inhibitor argatroban reduced macroscopic and histologic damage in TNBS-induced colitis rats. Furthermore, the specific HNE inhibitor silvelestat sodium hydrate (or ONO-5046) showed therapeutic effects in DSS-treated mice by significantly reducing weight loss and the histological score. It suppressed HNE activities in both the plasma and culture supernatant of colonic mucosa from DSS-induced colitis mice [120]. More recently, the chymase inhibitor TY-51469 has been shown to reduce experimental colitis in rats [121]. Other inhibitors have been tested in a cohort of patients with gut inflammation. The high specific and selective tryptase inhibitor APC-2059 has completed its phase II clinical trial in patients with mildly to moderately active UC [122]. This drug was well tolerated, and the patients displayed clinical improvement [122]. Moreover, nafamostat mesylate (or FUT-175), an extremely potent inhibitor of human tryptase [123] but not specific [124], has shown efficacy in reducing intestinal inflammation of rats with TNBS-induced colitis and human patients with colitis resistant to conventional therapy such as corticosteroids and sulfasalazine [125,126].

### 3.2. Natural Protease Inhibitors

#### 3.2.1. MMP Inhibitors

Considering the broad range of activities of MMP, tight regulation is required. One of the regulation mechanisms is provided by specific endogenous inhibitors named tissue inhibitors of metalloproteinases (TIMPs). In humans, the TIMPs family is composed of four members, namely TIMP-1, TIMP-2, TIMP-3 and TIMP-4, which are about 40% identical in sequence. Their inhibition property, uncovered by crystallographic structures, is mediated by the formation of a noncovalent complex in a 1:1 stoichiometry, where the N-domain of TIMP interacts with the catalytic site of MMPs. Indeed, a ridge formed by the conserved N-terminal motif Cys1-X-Cys3-X-X inserts into the MMP active site with Cys1 oriented in a position where its α-amino group and carbonyl group coordinate with the catalytic Zn^2+^ [127]. Although each TIMP can inhibit all MMPs, the specificity towards specific MMPs differ between each member such as, for example, TIMP-1 having reduced efficacy against some membrane-type MMPs (MMP-14, MMP-16, MMP-24). Interestingly, apart from interacting with mature MMPs, TIMPs also bind proenzymes through their C-domain, resulting in the activation or inhibition of the targeted MMP. The formation of the complex MMP-14–TIMP-2–pro-MMP-2 will lead to pro-MMP-2 activation in consequence of the cleavage of the propeptide by a free MMP14 [128]. On the contrary, the ternary complex between MMP-14–TIMP-4–pro-MMP-2 does not evoke MMP2 maturation caused by the efficient inhibition of MMP-14 by TIMP-4 [129]. TIMP expression is distributed over a wide range of tissues and appears to be either constitutive such as for TIMP-2 or inducible such as TIMP-1, -3, and -4. 

The balance between MMPs and TIMPs is crucial in the inflammation and tissue wound-healing processes, thus any disruption of it can elicit pathological processes by either favoriting the degradation of the ECM component via MMP activity or at the opposite provoking the accumulation of the ECM component and potential fibrosis. In IBD patients, increased levels of TIMP-1 were found in the cultured tissue of inflamed colonic mucosa biopsy, while undetectable levels were reported in uninflamed samples [130]. Some studies demonstrated TIMP-1 serum levels, in both CD and UC patients, were higher especially in active disease [131] and plasma levels in UC patients correlate positively with the disease activity [132]. Strikingly, a recent study suggests a role for TIMP-1 in the attenuation of inflammatory pain through MMP inhibition and receptor-mediated cell signaling [133]. No statistical difference for TIMP-2 serum levels was found between healthy and IBD patients as well as between patients with active or inactive disease [131]. Carbone et al. [134] suggested a reduction in TIMP-2 serum levels, under anti-TNF-α antibody treatment of IBD, to be used as a potential biomarker of short- and long-term remission. However, further investigations are required to clarify its role as an active pathophysiological factor. The implication of TNF-α in the disruption of the epithelial barrier is well established [135,136,137], as well as its enhanced level in CD patients. Active TNF-α results from the cleavage of the membrane-bound precursor by the TNF-α-converting enzyme (TACE or ADAM17), a protease submitted to TIMP-3 inhibition. Monteleone et al. [138] demonstrated TIMP-3 expression was downregulated in the mucosal biopsy from CD patients. Furthermore, TIMP-3-KO mice developed more severe colitis after TNBS administration than TIMP-3–transgenic mice [138]. Considering TIMP-4 serum levels, Kapsoritakis et al. [131] established that UC and CD patients had significantly lower levels than control.

#### 3.2.2. Serine Protease Inhibitors

Considering the importance of serine proteases in the maintenance of proteolytic homeostasis and their involvement in IBD [139], serine protease inhibitors, referred to as serpins, are recognized to play a key role in the proteolytic balance, which constitutes a potential therapeutic target [5,140]. Eukaryotic serpins were broadly studied in health and disease. With more than 12,953 genes encoding for eukaryotic serpins on the PubMed database until 2013 [141], and about 16,092 up to now, these inhibitors showed their tight implication in various physiological processes such as blood coagulation [142], hormone transport [143] and inflammatory responses [144]. In the case of IBD, a protease–antiprotease imbalance was reported with overexpression of serine proteases and an under-expression of their specific inhibitors [8,145]. Serpins are produced in the gastrointestinal tract either by intestinal epithelial cells or by infiltrated immune cells [146]. In pathological conditions, IBD patients displayed a disequilibrium of several serpins such as α-1-antitrypsin (SERPIN A1), an inhibitor of trypsin and chymotrypsin [147]. Studies revealed that α-1-antitrypsin is downregulated in IBD patients and that its administration to mice with intestinal inflammation could reduce inflammation and restore the epithelial barrier integrity [147]. Elafin, another natural serine protease inhibitor, produced by the intestinal epithelial cells, was shown to be underrepresented in mucosal surfaces of IBD patients [148]. Among its specificities, Elafin is able to inhibit human neutrophil proteases, notably elastase and proteinase 3. By deactivating those two proinflammatory proteases, Elafin contributes to the regulation of the inflammatory response. The same study indicated that the delivery of Elafin via Elafin-expressing bacteria to different mice models of acute and chronic intestinal colitis protected the gut homeostasis and prevented tissue damages [148]. Furthermore, a secretory leukocyte protease inhibitor (SLPI) is recognized to interfere in several protease-dependent pathways through its capacity to inhibit human trypsin, tryptase, catG and leucocyte elastase [149]. Therefore, SLPI was largely studied in an inflammatory context and appeared to be a potential candidate for IBD treatments [149]. Uncommonly, recent studies unveiled that the upregulation of *SERPIN1*/PAI-1 (plasminogen activator inhibitor 1) was correlated with inflammation severity and responsible for worsening colitis damages in mice [150]. Actually, PAI-1 is a specific inhibitor of the fibrinolytic protease tissue plasminogen activator (tPA), which is a key mediator of the anti-inflammatory transforming growth factor β (TGF-β) activation pathway. The study showed that PAI-1 enrichment exerts a proinflammatory role and exacerbated mucosal alteration [150].

Diet is an exogenous source of eukaryotic serine protease inhibitors, notably originating from plants. Indeed, plant serine protease inhibitors (PSPIs) are widely distributed among the plant kingdom [151], although most studied inhibitors belong to Leguminosae, Solanaceae and Graminae [152]. PSPI are classified into several groups: Bowman–Birk serine protease inhibitors, cereal trypsin/α-amylase inhibitors, mustard trypsin inhibitors, potato-type I inhibitors, potato-type II protease inhibitors, serpins, Kunitz inhibitors and squash inhibitors. Bowman–Birk inhibitors (BBIs), one of the most studied families of PSPI, are low-molecular-weight proteins (5–16 kDa), with two protein-binding sites localized at the opposite sides of the molecule, allowing the inhibition of two proteases independently [153]. The high disulfide content is mostly responsible for the high stability toward BBI extreme temperature and pH conditions including the GI environment [154,155]. In several animal studies, an anti-inflammatory effect has been associated with a soybean extract enriched BBI concentrate (BBIC) ingestion [156]. Furthermore, BBI is a potent inhibitor of human proteases released by inflammation-mediating cells, including catG [157], leukocyte elastase [158] and chymase [159], providing interest in its use as a potential IBD treatment. In DSS-induced colitis mice models, food supplementation with 0.5% BBIC leads to an improvement of the histopathological score, a lower mortality rate and a delay in the onset of mortality [160]. A low dose of fermented soy germ extract was shown to induce a decrease in fecal protease activity and PAR-2 expression due to BBI activity in a rat model of IBD [161]. In a pilot clinical trial, patients with active UC treated with BBIC showed DAI score improvement and induction of remission, although the results did not achieve statistical significance thus requiring further clinical trial investigation [162].

Compared to eukaryotic serpins, prokaryotic serpins remain poorly investigated. However, recent studies have given more interest to bacterial serpins, belonging essentially to the human gut microbiota, and their possible link with IBD [163]. The first reported bacterial serpin originates from an extremophilic bacteria [164]. Irving et al. studied the thermophilic bacterial serpin Thermopin, derived from *Thermobifida fusca*, characterized by its efficient inhibition towards the human chymotrypsin while maintaining high thermostability [164]. Tengpin, a novel bacterial serpin from *Thermoanaerobacter tengcondensis*, revealed the significant inhibition of HNE [165]. Taking into consideration the intriguing state of microbial dysbiosis in IBD patients, some reports shed the light on bacterial serpins from the human gut microbiota. These candidates disclosed an interesting potential to inhibit significantly a large spectrum of human serine proteases involved in IBD [166,167,168]. Mainly, we found Miropin, a serpin encoded by *Tanarella forsythia* [166], Siropin1 and Siropin2 from *Eubacterium sireaum* [167] and a serpin secreted by *Bifidobacterium longum* NCC2705 (SERPINBL) [168]. The latter showed its ability to inhibit HNE [168]. Regarding Siropin1 and Siropin2, it has been reported that they inhibit HNE and PR3, both known for their increased activity in IBD [167]. Siropins showed a more significant inhibition when compared to other serpins and were able to inhibit fecal proteases recovered from a DSS-induced colitis in a mice model [167]. Meanwhile, Miropin the serpin of *T. forsythia*, was characterized by a large spectrum of inhibition including serine proteases, for instance, trypsin, HNE, catG and papain cysteine protease [166]. It inhibits bacterial proteases as well, such as subtilisin and gingipain. The main challenge of targeting proteases associated with inflammation would be to identify a natural inhibitor with high specificity and stability and aim to restore the proteolytic equilibrium with fewer side effects compared to chemical compounds.

## 4. Conclusions

Serine proteases and MMPs are both involved in multiple biological processes such as digestion, immunity, wound healing and inflammatory response, together with their implication in maintaining GI homeostasis. Dysregulation of the proteolytic balance of these two major families of proteases has been linked to digestive inflammation. Studies investigating the potential interplay between MMPs and serine proteases are still in their infancy. More work is therefore needed to define these contributions in health and inflammation. Many advances have been made in developing selective, potent, metabolically stable inhibitors while minimizing their side effects. However, this task comes up against the complex interaction between those proteolytic enzymes and their inhibitors such as those between MMPs and TIMPs, which require further investigations to identify more specifically the proteases involved in the disease. In addition, proteases and protease inhibitors from the human gut microbiota are still poorly studied. Their characterization, despite its challenging aspect, might lead to uncovering new relevant targets for IBD treatment.

## Data Availability

Not applicable.
